# Stage-Specific Mucin Reprogramming Across the Gastric Cancer Cascade: Unveiling Molecular Mechanisms and Novel Therapeutic Vulnerabilities

**DOI:** 10.3390/biom16091307

**Published:** 2026-09-09

**Authors:** Xiao Dong, Xiaoyang Wu, Zeyu You, Kexin Lu, Huan Cai, Bo Zhang, Yuehua Gong, Qinchuan Wang, Yuan Yuan, Huakang Tu

**Affiliations:** 1 Center of Clinical Big Data and Analytics of the Second Affiliated Hospital, Zhejiang University School of Medicine, Hangzhou 310058, China; xdong3@zju.edu.cn (X.D.); 22418081@zju.edu.cn (Z.Y.); 22418061@zju.edu.cn (K.L.); 22418099@zju.edu.cn (H.C.); 12518175@zju.edu.cn (B.Z.); 2 Tumor Etiology and Screening Department of Cancer Institute and General Surgery, the First Hospital of China Medical University, Shenyang 110001, China; zdtkiu123@163.com (X.W.); yhgong@cmu.edu.cn (Y.G.); 3 Key Laboratory of Cancer Etiology and Prevention in Liaoning Education Department, the First Hospital of China Medical University, Shenyang 110001, China; 4 Key Laboratory of GI Cancer Etiology and Prevention in Liaoning Province, the First Hospital of China Medical University, Shenyang 110001, China; 5 Department of Big Data in Health Sciences, School of Public Health, Zhejiang University School of Medicine, Hangzhou 310058, China; 6 Department of Surgical Oncology, Affiliated Sir Run Run Shaw Hospital, Zhejiang University School of Medicine, Hangzhou 310058, China; wangqinchuan@zju.edu.cn; 7 Institute of Digital and Intelligent Health, Zhejiang University, Hangzhou 310058, China

**Keywords:** gastric cancer, glycosylation alterations, tumor microenvironment, therapeutic vulnerabilities

## Abstract

Mucins are a class of highly glycosylated macromolecules that constitute a cornerstone of the gastrointestinal mucosal barrier and play multiple essential roles in maintaining tissue homeostasis. During the progression from normal gastric mucosa through precancerous lesions to gastric cancer and metastasis, the expression profiles, glycosylation patterns, and spatial distribution of mucins undergo systematic and programmatic alterations, a process referred to as “mucin reprogramming.” Recent studies have revealed that this reprogramming is by no means a passive bystander phenomenon accompanying tumorigenesis; rather, it is a central biological event that actively drives malignant transformation, shapes an immunosuppressive microenvironment, and influences therapeutic response. This article aims to systematically delineate the dynamic landscape of mucin expression changes during gastric cancer progression, to provide an in-depth analysis of the underlying molecular mechanisms, and to focus on its clinical value and translational potential in the early diagnosis, molecular classification, prognostic assessment, and targeted therapy of gastric cancer. In addition, this review also discusses recent applications of artificial intelligence in the precise identification of mucin phenotypic features. By integrating the latest research advances, this review seeks to provide new perspectives and a theoretical basis for a deeper understanding of the mechanisms of mucin reprogramming during gastric cancer progression and for the development of novel diagnostic and therapeutic strategies.

## 1. Introduction

Gastric cancer is a leading cause of cancer-related mortality worldwide [1], and its molecular heterogeneity complicates the implementation of precision medicine [2]. Traditional classifications, such as the Lauren or WHO systems, describe static pathological features but do not fully capture the multistage process of tumor development. Investigating the molecular changes that occur during the onset and progression of gastric cancer, and identifying biomarkers and therapeutic targets associated with alterations in cell fate, may provide insights relevant for clinical applications.

The mucin family comprises high-molecular-weight glycoproteins secreted by epithelial cells or expressed on the cell membrane surface. In addition to functioning as lubricants or physical barriers, mucins play essential roles in cell signaling, immune regulation, and the establishment of the tumor microenvironment [3]. In normal gastric mucosa, mucin expression exhibits strict lineage specificity and spatial distribution patterns, and this regional distribution forms the basis for maintaining the differentiated state of gastric epithelial cells and tissue homeostasis [4]. In recent years, studies have shown that during tumor initiation and progression, the expression patterns of mucin genes, post-transcriptional modifications (particularly glycosylation), and functional states undergo profound changes, a phenomenon termed “mucin reprogramming” [5,6,7]. This reprogramming is highly ordered and stage-specific. It not only reflects shifts in the differentiation trajectory of epithelial cells, but also directly contributes to tumor cell proliferation, survival, invasion, immune evasion, and drug resistance through multiple mechanisms [8,9,10]. More recently, with the elucidation of mechanisms by which aberrant mucin glycosylation directly drives gastric carcinogenesis, together with deeper investigation into its interactions with the tumor microbiome, the role of mucins in gastric cancer biology has been elevated to a new level of understanding [11]. Therefore, a systematic analysis of mucin reprogramming during gastric cancer progression is of major clinical significance for the development of mucin-based approaches for early diagnosis, prognostic stratification, and novel targeted therapies.

Previous reviews have addressed mucin expression as a diagnostic or prognostic marker, mucin-associated O-glycosylation during gastric carcinogenesis, or selected pathogenic and therapeutic roles of gastric mucins and glycans [12,13,14,15]. However, these lines of evidence remain relatively fragmented across disease stages, molecular mechanisms, and clinical translation. The novelty of this review lies in proposing ‘mucin reprogramming’ as a stage-dependent integrative framework in which changes in mucin abundance, spatial lineage identity, and glycosylation are treated as dynamically coordinated phenotypes that evolve across disease stages rather than as isolated, static markers. This framework links these phenotypic changes to upstream drivers and downstream clinical applications, including risk stratification, molecular classification, glycotope-targeted therapy, and artificial intelligence-assisted phenotyping. By highlighting when specific phenotypes emerge, how they are maintained, and how they may inform individualized clinical decisions, this integrative perspective addresses a gap in prior reviews, which have generally not examined the dynamic evolution of mucin phenotypes together with their clinical integration.

Within this framework, this review systematically delineates dynamic changes in mucin expression across the continuum from chronic inflammation, atrophy, and intestinal metaplasia to malignant transformation, invasion, and metastasis. It further analyzes microbiota-, epigenetic-, and glycosylation-related mechanisms that drive mucin reprogramming and the biological consequences of this process. On this basis, the review summarizes the clinical and translational relevance of mucin reprogramming for early diagnosis, molecular subtyping, prognostic assessment, and targeted therapy in gastric cancer. Moreover, it discusses how emerging technologies, including artificial intelligence, may integrate multi-omics data and translate microscopic mucin phenotypes into quantitative features for prediction and clinical decision support, thereby providing new perspectives and a theoretical foundation for precision diagnosis and treatment of gastric cancer.

## 2. Structural Classification and Functional Roles of the Mucin Family

**Figure 1:**
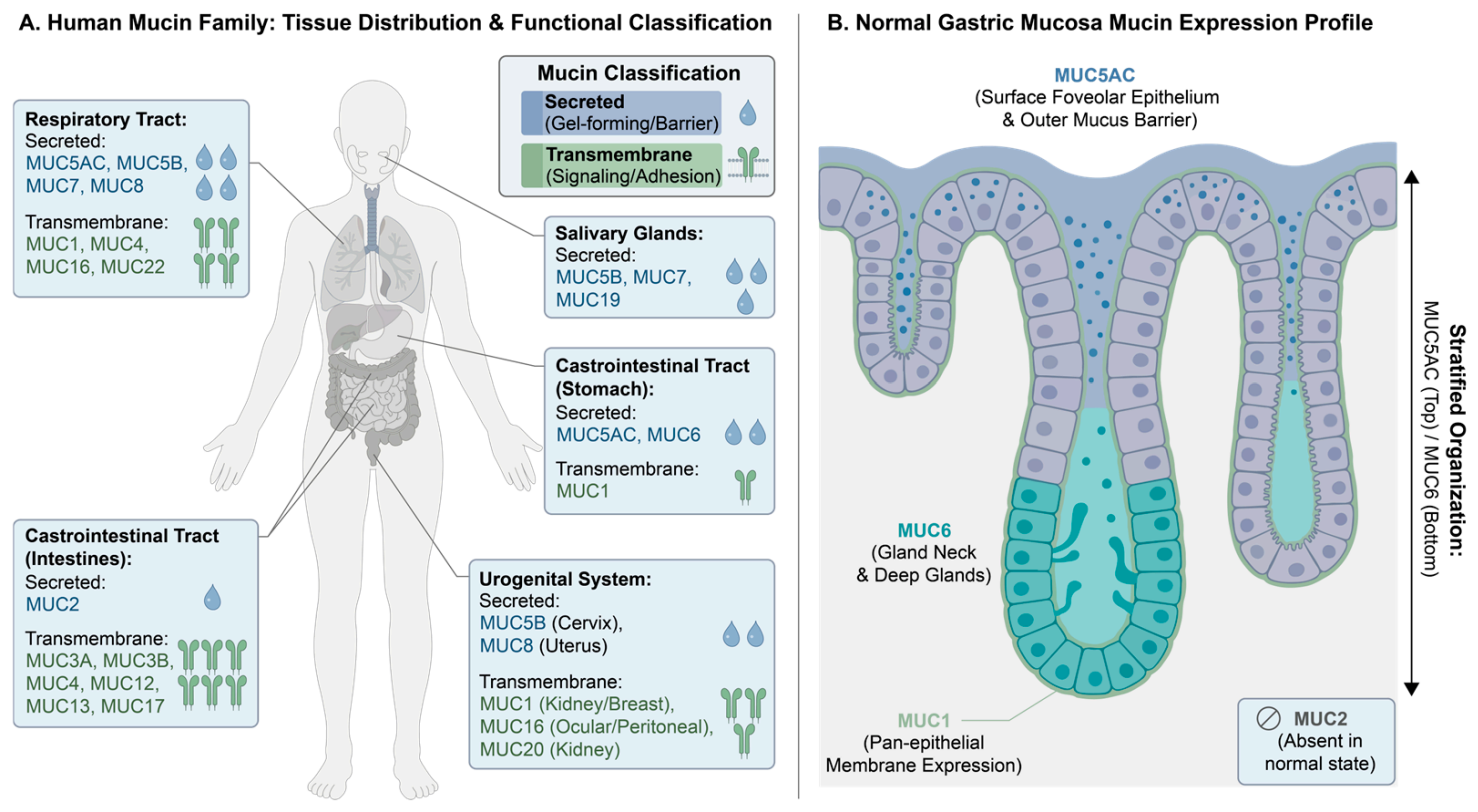
Overview of the tissue distribution of the human mucin family and the mucin expression landscape in normal gastric mucosa. (**A**) Schematic illustration of the tissue distribution and functional classification of representative secreted and transmembrane mucins. (**B**) Compartment-specific mucin expression in normal gastric mucosa, with MUC5AC in the surface and foveolar epithelium, MUC6 in the mucous neck and deep gland compartments, and MUC1 along epithelial cell membranes; MUC2 is normally absent.

The human mucin family is a cluster of structurally diverse and functionally specialized glycoproteins, with at least 22 members identified to date (Table 1). According to their molecular structure and membrane-anchoring properties, they can be classified into two major categories, namely secreted mucins and transmembrane mucins. Together, these two groups form the molecular basis of epithelial defense and cell communication. Secreted mucins, mainly including MUC5AC, MUC6, MUC2, MUC5B, MUC7, and MUC19, are characterized by their ability to form extracellular mucus gel networks through polymerization. These mucins contain tandem repeat sequences rich in serine, threonine, and proline, which provide sites for O-glycosylation [16]. The resulting mucus layer forms a physicochemical barrier that protects the epithelial surfaces of the respiratory, gastrointestinal, and reproductive tracts. Mucins help lubricate and hydrate these surfaces and trap pathogens and particulate matter [17]. Transmembrane mucins, such as MUC1, MUC4, MUC13, MUC16 (CA125), as well as MUC3A/B, MUC12, and MUC17, are glycoproteins localized on the cell surface and characterized by a single-pass transmembrane domain and a cytoplasmic tail. They participate in intercellular adhesion and recognition, and also function as important signal transduction molecules and cell-surface sensors [12]. The extracellular domains detect changes in the microenvironment, while the intracellular tails interact with signaling molecules to influence cell proliferation, differentiation, apoptosis, and immune responses. Under pathological conditions, especially during tumorigenesis, the expression levels, glycosylation patterns, and spatial distribution of transmembrane mucins often undergo marked alterations, thereby driving malignant phenotypes and influencing the tumor microenvironment [13]. In summary, members of the mucin family, by virtue of their unique structural properties, perform highly coordinated and specialized functions under physiological conditions to maintain mucosal homeostasis; dysregulation of their expression and function, in turn, becomes a key event in the development and progression of multiple diseases, particularly epithelial-derived tumors (Figure 1A).

## 3. Dynamic Landscape of Mucin Expression During Gastric Cancer Progression

### 3.1. Mucin Expression Characteristics in Normal Gastric Mucosa

**Figure 2:**
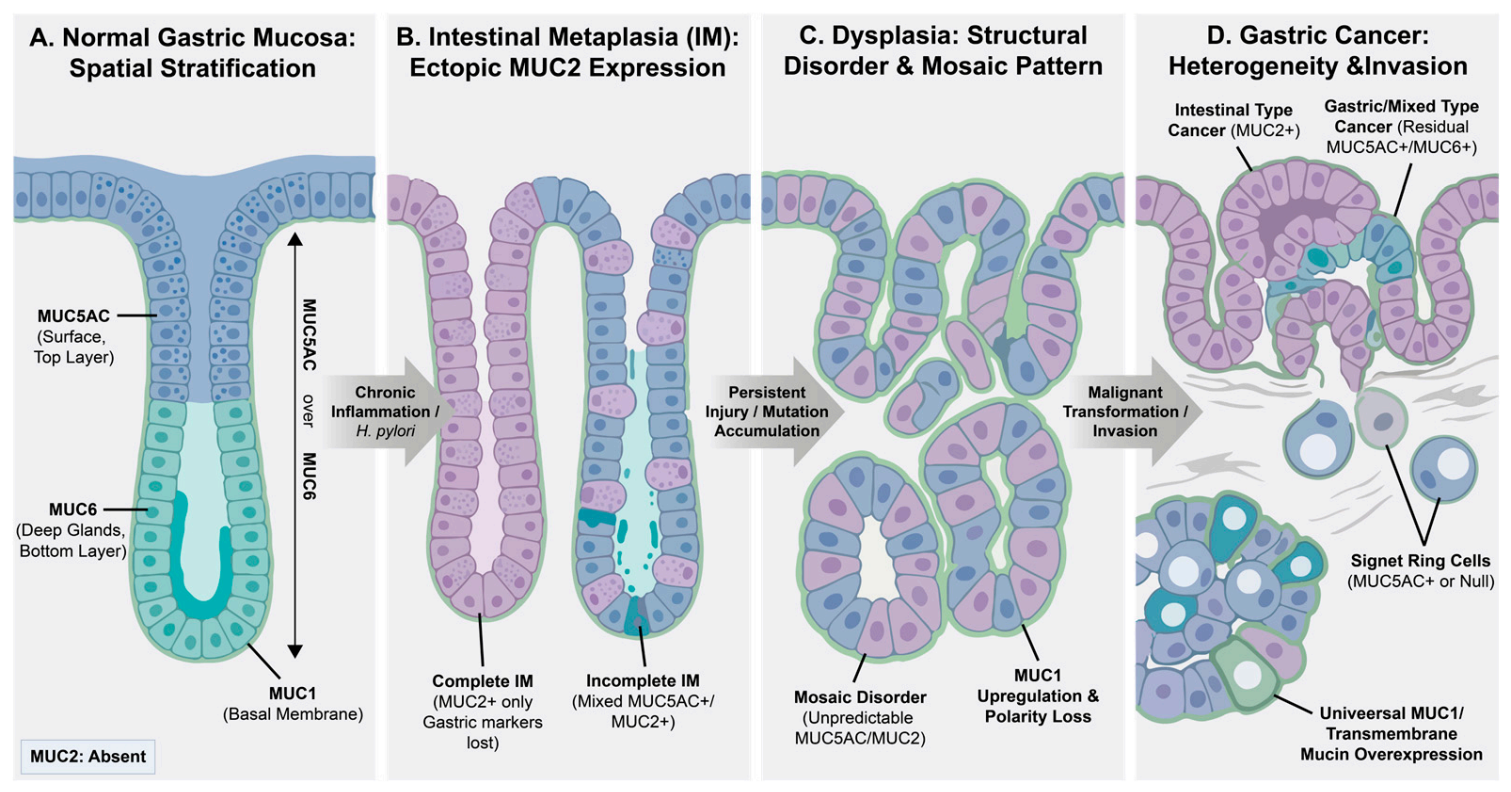
Dynamic reprogramming of mucins across the Correa cascade in gastric mucosa. (**A**) Normal gastric mucosa shows spatially segregated MUC5AC and MUC6 expression, epithelial MUC1 expression, and absence of MUC2. (**B**) Intestinal metaplasia is characterized by acquired MUC2 expression; complete intestinal metaplasia is characterized by loss of gastric-type markers, whereas incomplete intestinal metaplasia retains mixed MUC5AC/MUC2 expression. (**C**) Dysplasia displays disorganized glandular architecture, mosaic mucin expression, MUC1 upregulation, and loss of epithelial polarity. (**D**) Gastric cancer shows heterogeneous gastric, intestinal, mixed, or null mucin phenotypes, together with invasive growth and increased transmembrane mucin expression. Arrows indicate the proposed direction of disease progression.

The mucins distributed in human gastric mucosa mainly include the secreted gel-forming mucins MUC5AC and MUC6, as well as the transmembrane mucin MUC1, and their expression patterns exhibit strict cell type specificity and spatial regionality [34]. MUC5AC is mainly expressed in surface epithelial cells and gastric foveolar cells, forming the first line of defense against external insults. MUC6 is mainly expressed in mucous neck cells and glandular cells in the deep part of the gastric glands, protecting the glandular structure and participating in the regulation of the gastric gland microenvironment. Together, they establish a complementary barrier pattern characterized as “MUC5AC above and MUC6 below” [35]. MUC1 shows a basal and widespread expression pattern on the surface membrane of epithelial cells, whereas the intestinal-type mucin MUC2 is completely absent under normal physiological conditions [10,18]. This classical “MUC5AC+/MUC6+/MUC2−” expression profile constitutes the molecular basis for the normal differentiation, homeostatic maintenance, and functional activity of gastric mucosal epithelial cells, and also provides a critical reference framework for identifying “mucin reprogramming” under pathological conditions (Figure 1B and Figure 2A).

### 3.2. Mucin Expression Characteristics in Gastric Mucosa with Intestinal Metaplasia

At the stage of intestinal metaplasia (IM), the gastric mucosal epithelium undergoes a critical phenotypic transition from a gastric type to an intestinal type [36]. The mucin expression pattern shows systematic reprogramming, with the most prominent hallmark being the ectopic acquired expression of the intestinal-type secretory mucin MUC2 [35,37], accompanied by a marked downregulation or loss of the originally dominant gastric-type mucins MUC5AC and MUC6. According to the expression pattern of mucins, this stage can be further divided into complete and incomplete types: complete intestinal metaplasia is characterized by a fully intestinal phenotype of MUC5AC−/MUC6−/MUC2+, whereas incomplete intestinal metaplasia is characteristically defined by the coexistence of gastric-type and intestinal-type mucins, such as co-expression of MUC5AC+ and MUC2+ [37,38,39,40]. In recent years, CD10, a marker expressed on the brush border of the intestinal epithelium and a specific hallmark of the small intestinal brush border, has been found to exhibit strong linear expression along the brush border in complete intestinal metaplasia glands, whereas its expression is absent or markedly attenuated in incomplete IM [41]. The incorporation of CD10 immunohistochemistry into traditional mucin phenotyping enables further subclassification of IM into two functional subgroups—“CD10-positive complete type” and “CD10-negative incomplete type”—thereby providing a new dimension for precise IM subtyping and offering a more objective molecular basis for clinical IM risk stratification and the formulation of endoscopic surveillance strategies. In addition, the expression pattern of the transmembrane mucin MUC1 during the IM stage exhibits a downregulation trend similar to that observed for the aforementioned secretory mucins [35]. In summary, the disordered mucin expression profile at the IM stage signifies a profound abnormality in cellular identity recognition and is closely associated with an elevated risk of gastric cancer progression (Table 2, Figure 2B).

### 3.3. Mucin Expression Characteristics in Dysplastic Gastric Mucosa

At the stage of gastric mucosal dysplasia, the mucin expression pattern evolves from the orderly phenotypic transition seen in intestinal metaplasia into a highly disordered and markedly heterogeneous aberrant pattern. On the one hand, the original region-specific expression architecture is completely disrupted, giving rise to a “mosaic”-like disordered landscape characterized by the regional loss of the intestinal-type mucin MUC2 together with the unpredictable reappearance of the gastric-type mucins MUC5AC and MUC6, indicating a complete loss of control over the cellular differentiation program [40,42,43]. On the other hand, the expression of the transmembrane mucin MUC1, which is closely associated with the acquisition of malignant potential, is significantly upregulated [10,44], while mucin molecules generally exhibit tumor-associated aberrant glycosylation modifications, such as the Tn and sialyl-Tn antigens [45,46]. This combination of disordered expression patterns, aberrantly high MUC1 expression, and premature glycosylation abnormalities represents a key molecular manifestation of clonal epithelial proliferation, loss of polarity, and activation of proliferative signaling pathways, and is directly associated with a very high immediate risk of malignant transformation. This is particularly evident in high-grade dysplasia, providing a core molecular basis for the pathological identification and risk stratification of such lesions (Figure 2C).

### 3.4. Mucin Expression Characteristics in Gastric Cancer Tissues

Following the progression to gastric cancer, mucin expression in the gastric mucosa exhibits systematic reprogramming and pronounced heterogeneity. Overall, secretory mucins display a loss of the gastric phenotype, characterized by marked downregulation or absence of MUC5AC and MUC6, accompanied by ectopic acquisition of the intestinal mucin MUC2 in a subset of gastric cancer tissues [27,47]. In contrast, transmembrane mucins are often upregulated in gastric cancer. High levels of MUC1, MUC4, MUC13, and MUC16 have been linked to tumor proliferation, invasion, metastasis, and poorer prognosis [27,30]. Based on the expression of secretory mucins, gastric cancer tissues can be classified into different mucin phenotypes: gastric (MUC5AC+/MUC6+), intestinal (MUC2+), mixed (co-expression of gastric and intestinal mucins), and null (lacking these mucins). Within the Lauren classification, intestinal-type gastric cancer typically shows an intestinal mucin profile, with high MUC2 expression and frequent nuclear positivity for CDX2, consistent with malignant transformation from intestinal metaplasia. In contrast, diffuse-type gastric cancer mainly corresponds to the gastric or null phenotype, with retention of partial MUC5AC/MUC6 expression or complete loss thereof, which is closely associated with diffuse infiltration, poor cohesive growth, and suboptimal therapeutic response characteristic of diffuse-type carcinoma [48]. In summary, the mucin expression signature in gastric cancer tissues represents a systematic reprogramming process, transitioning from “loss of gastric identity” to “acquisition of intestinal/aberrant transmembrane features”. This highly heterogeneous expression pattern not only constitutes an integral component of the molecular classification of gastric cancer but may also serve as a potential prognostic indicator and a target for the development of targeted therapies [49,50], thereby providing key molecular insights for precision diagnosis, prognostic evaluation, and targeted therapy of gastric cancer [47,50] (Figure 2D).

## 4. Driving Mechanisms of Mucin Reprogramming and Their Synergistic Effects

The transition of mucins from physiological defense to pathological mediation during gastric cancer progression is the result of systematic reprogramming orchestrated by a multidimensional molecular network. This process is mainly driven by the coordinated action of three core mechanisms: microbiome dysbiosis, epigenetic dysregulation, and aberrant glycosylation.

### 4.1. Microbiome Dysbiosis

**Figure 3:**
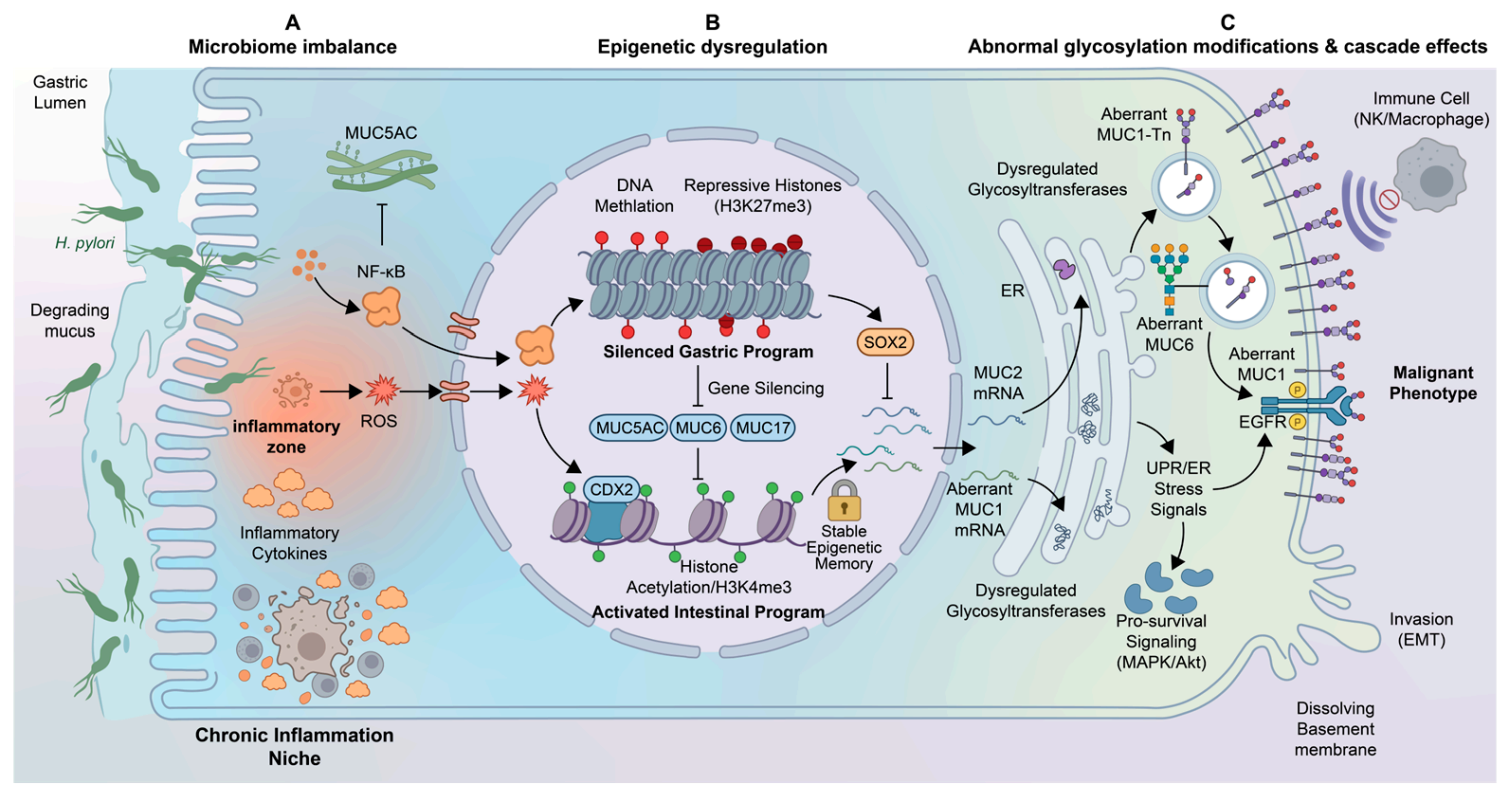
The coordinated action of the three core mechanisms drives mucin reprogramming and its cascade effects. (**A**) Microbiome dysbiosis and Helicobacter pylori infection disrupt the mucus barrier and activate NF-κB, reactive oxygen species, and inflammatory cytokines. (**B**) Altered DNA methylation and histone modifications suppress gastric-lineage programs and promote CDX2-associated intestinal differentiation. (**C**) Dysregulated glycosylation generates aberrant mucin glycoforms, endoplasmic-reticulum stress, pro-survival signaling, immune evasion, and epithelial–mesenchymal transition.

From the perspective of microenvironmental mechanisms driving mucin reprogramming, the “microbiome–mucin” axis constitutes a powerful microecological driving engine. Its core mechanism lies in the fact that the microbiome, as an upstream initiating and persistent driving factor, actively rewrites the expression and functional programs of mucins through multilevel actions. A representative example is Helicobacter pylori, which directly activates inflammatory signaling pathways such as NF-κB in host cells through its virulence factors, thereby inducing the expression of the oncogenic transmembrane mucin MUC1 and suppressing the protective mucin MUC5AC, achieving initial reprogramming at the molecular level [11,51]. Furthermore, the chronic inflammatory microenvironment triggered by microbial colonization drives epigenetic alterations such as DNA methylation in mucin genes through mediators including reactive oxygen species, and affects glycosyltransferase expression to induce aberrant glycosylation, thereby stabilizing reprogramming at the level of molecular modification. For example, *H. pylori* infection can induce hypermethylation of the MUC17 gene promoter, leading to silencing of this anti-inflammatory transmembrane mucin and thereby further amplifying the inflammatory response [30]. In addition, carcinogenic metabolites produced by certain bacteria can directly damage the epithelium and shift the expression profile toward a malignant phenotype. Importantly, reprogrammed mucins, such as aberrantly glycosylated MUC1, in turn create a favorable ecological niche for the enrichment of specific microbial communities by altering their physicochemical properties, for example, by providing abnormal glycan receptors or changing nutrient availability. These reshaped microbial communities then impose new selective pressures, thereby forming a self-reinforcing loop of “microbiota-driven reprogramming → reprogramming-reshaped microbiota → newly reshaped microbiota-driven reprogramming” [52]. This loop jointly drives the establishment of chronic inflammation and an immunosuppressive microenvironment, thereby not only promoting malignant transformation but also profoundly affecting therapeutic response [53]. Therefore, this bidirectional interaction is essentially a key mechanism by which microecological factors actively drive and continuously amplify mucin reprogramming, providing a theoretical basis for strategies aimed at interrupting this driving loop, ranging from antimicrobial treatment and microbiome intervention to combination therapy (Figure 3A).

### 4.2. Epigenetic Dysregulation

As the “programmatic instruction system” driving mucin reprogramming, epigenetic regulation systematically rewrites chromatin states and gene accessibility without altering the DNA sequence, thereby enabling lineage switching of transcription factor programs, such as ectopic activation of intestinal transcription factor CDX2 and silencing of gastric transcription factor SOX2, and exerting precise spatiotemporal on-off control over mucin genes. In this way, it dominates the malignant identity transition of gastric epithelial cells. This driving network encompasses three coordinated levels. First, DNA methylation functions as a “master switch” for gene silencing, whereby hypermethylation of CpG islands in the promoter regions of specific mucin genes directly leads to transcriptional silencing. For example, MUC2 is silenced by methylation in normal gastric mucosa, and its demethylation during the stage of intestinal metaplasia is a critical driving step for its ectopic activation; conversely, hypermethylation of the gastric-type mucins MUC5AC and MUC6 during gastric cancer progression directly drives the loss of the gastric phenotype. Second, histone modifications act as “fine-tuners” of chromatin state by dynamically regulating covalent modifications, such as activating H3K4 trimethylation and acetylation or repressive H3K27 trimethylation, thereby controlling transcription factor binding and cooperating with DNA methylation to establish stable “epigenetic memory.” Third, non-coding RNAs, such as miR-145, which directly targets and suppresses MUC2, function as “upstream conductors” of the expression network by exerting rapid and precise effects through post-transcriptional regulation [54,55,56,57]. These mechanisms do not operate independently; rather, they act in precise coordination to drive stage-specific reprogramming. During intestinal metaplasia, transcription factors such as CDX2 can recruit demethylases and histone acetyltransferases to the MUC2 locus, promoting its demethylation and chromatin opening, while simultaneously initiating repressive modifications at gastric-type loci, thereby programmatically driving the transition from a “gastric type” to an “intestinal type.” At the stages of dysplasia and carcinogenesis, epigenetic dysregulation is further intensified under signals such as chronic inflammation, driving the silencing of tumor-suppressive mucins, such as MUC5AC, and the activation of oncogenic mucins, such as MUC1, thereby promoting the acquisition of a fully malignant phenotype. In summary, epigenetic regulation provides order, plasticity, and memory for mucin reprogramming and serves as the central bridge connecting environmental factors with changes in gene expression. Its translational significance lies in the fact that specific methylation markers of mucin genes may serve as potential liquid biopsy biomarkers, whereas agents targeting demethylation or histone deacetylase inhibitors may theoretically offer new strategies to “reverse” aberrant reprogramming and intervene in precancerous lesions (Figure 3B).

### 4.3. Aberrant Glycosylation

Profound dysregulation of glycosylation is a core mechanism driving mucin reprogramming in gastric cancer. Its role extends far beyond that of a passive post-translational modification; rather, it acts as a critical “code” that determines the functional activity of mucins. This abnormality actively drives malignant transformation through three hierarchical steps. At the initiation stage, dysregulated expression of core glycosyltransferases directly leads to “source errors” in O-glycosylation. At the elongation stage, disruption of key enzymes causes “programmatic disorder” in glycan synthesis, thereby impairing the mucin gel network and its barrier function. At the terminal modification stage, aberrant overexpression of enzymes such as sialyltransferases leads to “identity masking,” exposing tumor-associated glycan antigens such as sialyl-Lewis X/A, which directly drive immune evasion and metastasis. This systematic glycosylation defect promotes tumor progression through multiple pathways. It directly compromises the integrity of the mucosal barrier. More importantly, recent studies have shown that glycosylation defects in gastric mucins can trigger endoplasmic reticulum-Golgi stress, which is then hijacked by cancer cells to aberrantly activate pro-survival signaling pathways such as MAPK/ERK, thereby converting a molecular defect into a sustained driving force for malignant transformation. At the same time, abnormal glycans can function as “don’t eat me” signals by binding to inhibitory receptors on immune cells, thereby reshaping the immunosuppressive microenvironment [45,58,59,60,61]. Therefore, aberrant glycosylation is a central driving force and amplifier throughout the entire reprogramming process, and intervention strategies targeting specific glycosylation enzymes or aberrant glycans provide a highly promising therapeutic direction for “correcting” reprogramming at the molecular level (Figure 3C).

### 4.4. Synergistic Effects and Cascade Events of the Three Core Driving Mechanisms

During mucin reprogramming in gastric cancer, the three core driving mechanisms—microbiome dysbiosis, epigenetic dysregulation, and aberrant glycosylation—operate in a sequential yet interconnected manner, transmitting upstream perturbations into downstream phenotypic alterations. Microbiome dysbiosis, particularly persistent Helicobacter pylori colonization, serves as the initial environmental trigger, imposing sustained inflammatory and oxidative stress on gastric epithelial cells through bacterial toxins and metabolites [62,63]. This disruption of mucosal homeostasis creates a microenvironment that primes subsequent molecular changes. Within this inflammatory context, epigenetic mechanisms can remodel the chromatin landscape of mucin genes. Promoter demethylation and permissive histone modifications may contribute to activation of intestinal-type mucin genes such as MUC2 [54,55]. Ectopic MUC2 expression changes the biochemical composition of the gastric mucus layer and accompanies acquisition of an intestinal epithelial program. Current evidence therefore supports interpreting MUC2 induction as part of a broader lineage-reprogramming process, rather than as a proven independent driver of malignant transformation. Conversely, epigenetic silencing may contribute to transcriptional downregulation of gastric-type mucins, including MUC5AC and MUC6 [64]. Loss of these protective mucins weakens the mucus barrier and is associated with disruption of glandular architecture and progression toward metaplasia and malignancy. Epigenetic regulation may therefore stabilize environmentally induced changes in gastric epithelial identity. Concurrently, dysregulated glycosyltransferase activity remodels mucin O-glycans [58,59]. This process can expose truncated tumor-associated glycotopes and impair mucus barrier function. Experimental models show that loss of core 1-derived O-glycans disrupts gastric epithelial homeostasis and promotes gastritis and gastric cancer, whereas MUC6 deficiency or defective gastric mucin glycosylation induces Golgi stress, MAPK activation, and aberrant glycan expression [46,58]. Together, these findings support a mechanistic link among mucin and glycan defects, inflammatory signaling, and gastric tumorigenesis.

These changes—ectopic MUC2 expression, downregulation or loss of gastric-type mucins, and truncation or remodeling of mucin glycans—may converge to destabilize mucosal homeostasis, promote inflammation and pro-tumorigenic signaling, and facilitate progression toward invasive cancer. Moreover, the resulting compromised barrier and altered glycocalyx feed back to the microbiome, favoring more invasive microbial communities, which in turn reinforce epigenetic and glycosylation abnormalities [65]. Thus, the cascade constitutes a self-perpetuating loop. Clinically, this implies that interventions at the precancerous stage should target upstream triggers such as H. pylori eradication, whereas established cancers may require combinatorial strategies that restore protective mucins and glycans while interrupting epigenetic and glycosylation-driven oncogenic signaling, including that associated with ectopic MUC2 expression.

## 5. Clinical Translational Applications of Mucin Reprogramming

Mucin reprogramming has potential clinical applications across multiple stages of gastric cancer management. At the precancerous stage, it may aid in risk assessment and early diagnosis. In advanced disease, it can inform targeted treatment strategies. Following treatment, it may contribute to prognostic evaluation and dynamic monitoring. The molecular changes identified through mucin reprogramming thus provide information relevant to key clinical decisions. This approach moves beyond static morphological assessment, extending pathological evaluation to dynamic molecular functional pathology, and offers a framework for risk stratification, precision therapy, and prognostic assessment in gastric cancer.

### 5.1. Early Screening and Early Diagnosis

**Figure 4:**
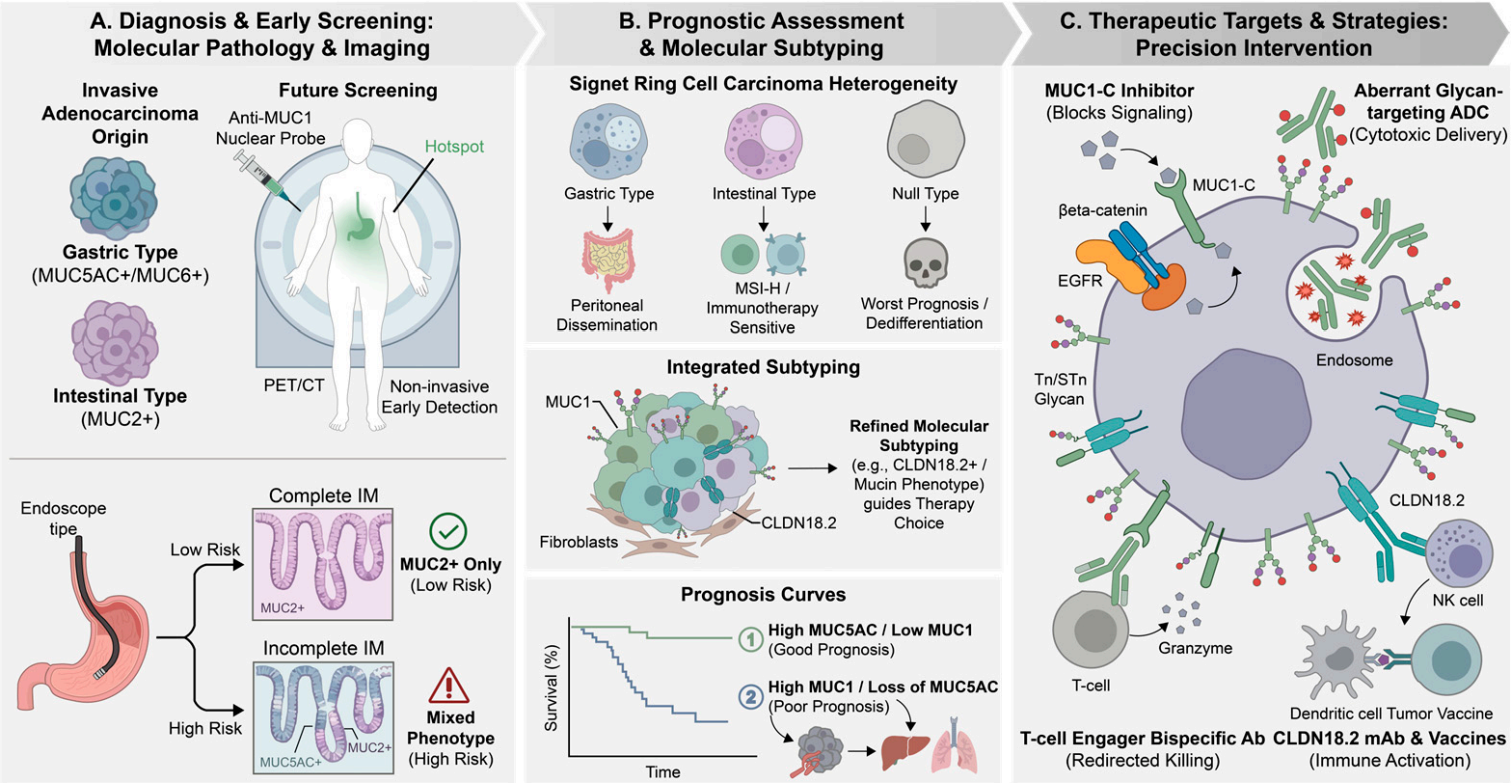
Clinical translational applications of mucin reprogramming in gastric cancer. (**A**) Mucin phenotyping may help distinguish completely from incomplete intestinal metaplasia, support cancer-risk assessment, complete tumor differentiation, and facilitate the development of MUC1-directed molecular imaging. (**B**) Gastric, intestinal, and null mucin phenotypes provide complementary information on tumor heterogeneity, metastatic patterns, and prognosis and may be integrated with biomarkers such as CLDN18.2 for refined patient stratification. (**C**) Potential therapeutic strategies include MUC1-C inhibitors, aberrant glycan-targeting antibody–drug conjugates, T-cell-engaging bispecific antibodies, tumor vaccines, and CLDN18.2-directed agents.

In the screening and management of precancerous lesions and the early diagnosis of gastric cancer, the mucin expression profile has become an indispensable molecular yardstick. Based on the immunohistochemical combination of MUC2, MUC5AC, and MUC6, complete and incomplete intestinal metaplasia can be clearly distinguished: single positivity for MUC2 with complete absence of gastric-type mucins indicates low-risk complete metaplasia; in contrast, a “mixed phenotype” in which MUC2 and MUC5AC are co-expressed in the same gland or cell specifically indicates disordered differentiation regulation and incomplete metaplasia associated with a significantly increased risk of malignant transformation, thereby providing a critical basis for precise endoscopic surveillance [35,40]. In addition, in the diagnosis of invasive adenocarcinoma, the mucin phenotype helps determine the histogenetic origin of tumor tissue and distinguish intestinal-type gastric cancer arising in a metaplastic background (MUC2+) from gastric-type gastric cancer arising from native glands (MUC5AC+/MUC6+). This is of important value for understanding the pathogenic mechanisms of different molecular subtypes and for formulating adjuvant treatment strategies [47,48]. A study of 127 patients with early gastric cancer showed that the combined use of magnifying endoscopy and markers such as MUC2, MUC5AC, and MUC6 achieved significantly higher diagnostic sensitivity and specificity than any single method, thereby realizing a complementary advantage between morphology and functional molecular pathology [66]. More advanced translational studies have begun to explore the application of mucin targets in in vivo imaging. An ongoing clinical study has registered a radionuclide probe based on an anti-MUC1 antibody, aiming to achieve early and precise visual diagnosis of gastric cancer by PET/CT. This marks the transition of mucin targets from pathological sections to whole-body molecular imaging and offers revolutionary prospects for noninvasive early screening [67,68] (Figure 4A).

### 5.2. Molecular Classification and Prognostic Assessment

Mucin phenotypes reflect the biological heterogeneity of gastric cancer and can be used for molecular classification and outcome prediction. Based on mucin expression, gastric cancer can be divided into several subgroups. The “gastric-type” subgroup retains MUC5AC expression and shows a tendency toward peritoneal dissemination. The “intestinal-type” subgroup expresses MUC2 and is frequently associated with microsatellite instability (MSI), which may increase sensitivity to immunotherapy. The “null” phenotype, characterized by complete loss of mucin expression, shows the highest level of dedifferentiation and invasiveness and is linked to poorer prognosis [53,69]. Recent studies have found that CLDN18.2-positive tumors are often associated with specific mucin phenotypes (such as the diffuse type) and a unique tumor microenvironment (such as stromal-rich and immunosuppressive features) [50,70]. This suggests that combining mucin-based classification with detection of targets such as CLDN18.2 can help establish a more refined molecular classification system, thereby providing a basis for selecting individualized treatment strategies centered on chemotherapy, immunotherapy, or targeted therapy. A meta-analysis including 28 studies and 4603 patients confirmed that high MUC1 expression was significantly associated with greater depth of tumor invasion, lymph node metastasis, vascular invasion, and poorer 5-year survival; similarly, loss of MUC5AC expression also indicated poor prognosis. This established the value of MUC1 and MUC5AC as independent prognostic markers [47]. This prognostic value is particularly evident in signet ring cell carcinoma, a highly heterogeneous subtype. Therefore, incorporating mucin phenotypes into routine pathological reports can provide a molecular basis for developing differentiated follow-up strategies and preventive approaches for peritoneal metastasis (Figure 4B).

As an established reference framework, The Cancer Genome Atlas (TCGA) classifies gastric cancer into four molecular subtypes—Epstein–Barr virus (EBV)-positive, microsatellite instability (MSI), genomically stable (GS), and chromosomal instability (CIN)—based on genomic and etiological features [14,71]. Although these subtypes capture major genomic and etiological variation, they do not directly resolve epithelial lineage, differentiation status, spatial heterogeneity, or glycosylation patterns. Mucin-based profiling could therefore provide complementary phenotypic information, help resolve heterogeneity within genomic subtypes, and identify surface glycotopes relevant to diagnosis and therapy [14,15]. Integrating mucin phenotypes with TCGA classification may thus improve biological interpretation, prognostic stratification, and clinical decision-making regarding mucin- or glycan-targeted interventions.

### 5.3. Therapeutic Targets and Precision Intervention Strategies

Small-molecule inhibitors targeting the cytoplasmic tail of the transmembrane mucin MUC1 (MUC1-C) represent a recent advance in blocking oncogenic signaling. These agents directly disrupt the interaction of MUC1-C with β-catenin or EGFR, thereby blocking sustained activation of the Wnt and MAPK pathways and reversing tumor stemness and metabolic reprogramming [72]. The conceptual innovation of this strategy lies in targeting a mucin-derived intracellular signaling node rather than a single canonical kinase, with the potential to disrupt multiple oncogenic pathways and exploit dependence on MUC1-C signaling. In the field of antibody-based therapeutics, antibody-drug conjugates (ADCs) targeting tumor-specific glycosylated epitopes, such as the Tn or STn antigens, have shown considerable potential. These agents use aberrantly glycosylated mucins as carriers for receptor-mediated endocytosis to deliver cytotoxic payloads into cancer cells, thereby reducing damage to normal tissues [6,73]. Importantly, Tn and STn are truncated O-glycan structures with limited exposure on most normal epithelia but increased expression during malignant transformation, making them cancer-associated post-translational targets. Beyond ADCs, several strategies are being explored to target aberrant glycans. CAR T cells engineered to recognize the Tn-MUC1 glycoform have demonstrated preclinical activity in adenocarcinoma models, providing a rationale for evaluation in gastric cancer [73]. Experimental modulation of glycosyltransferases is also being investigated to alter the biosynthesis of tumor-associated glycans, although target specificity and toxicity remain important challenges [59]. Sialidase-based and other glycocalyx-editing approaches aim to remove immunosuppressive sialic acid residues and enhance antitumor immune responses [74,75]. In addition, lectin–drug conjugates can recognize tumor-associated glycan patterns and deliver cytotoxic payloads; preclinical activity has been demonstrated in glycan-defined gastric cancer models [46]. At the same time, bispecific antibody technology has reshaped immunotherapeutic strategies. Novel bispecific antibodies can simultaneously bind MUC1 on the tumor surface and CD3 on T cells, thereby redirecting T cell-mediated cytotoxicity through a bridging effect [76,77]. In addition, tumor vaccines targeting aberrant MUC1 glycopeptides are designed to break immune tolerance and activate adaptive immune responses through the presentation of specific antigens [6,78]. These strategies mark a shift in gastric cancer treatment from nonspecific chemotherapy toward a precision-targeted model based on mucin biology.

In recent years, CLDN18.2, as a gastric-specific target closely associated with mucin biology, has achieved a milestone breakthrough. A monoclonal antibody targeting CLDN18.2 in combination with chemotherapy has now been approved for first-line treatment based on the results of a global phase III clinical trial, significantly improving survival in patients with CLDN18.2-positive, HER2-negative advanced gastric cancer [50,79]. In a phase I clinical trial of the CLDN18.2-targeted ADC SHR-A1904, a topoisomerase I inhibitor was used as the payload. Because this agent does not show cross-resistance with commonly used chemotherapeutic drugs, it still demonstrated an encouraging objective response rate and survival benefit in refractory patients who had received multiple prior lines of therapy, including some who had previously undergone CLDN18.2-targeted treatment, thus providing a new strategy for overcoming drug resistance [80,81] (Figure 4C).

In summary, the clinical translation of mucin reprogramming has developed into a complete application system encompassing risk warning, early diagnosis, targeted therapy, and prognostic assessment. Current research frontiers lie not only in identifying new targets, but also in integrating multiple targets, such as the MUC profile, CLDN18.2, and PD-L1, into a unified classification framework to guide combination treatment strategies, as well as in using new technologies such as ADCs, bispecific antibodies, glycan-editing enzymes, and glycosyltransferase inhibitors to overcome drug resistance. The therapeutic strategies discussed here extend beyond conventional protein-directed approaches to include aberrant glycosylation patterns and mucin-derived intracellular signaling, thereby broadening the range of potentially targetable dependencies in gastric cancer. In the future, with continued advances in mucin-based molecular imaging, liquid biopsy, and more intelligent clinical trials of combination therapies, the precision diagnosis and treatment system for gastric cancer guided by mucin reprogramming will become increasingly mature.

## 6. Precise Identification of Mucin Phenotypes Empowered by Artificial Intelligence

Although mucin phenotypes have clear clinical value in risk stratification for gastric cancer, their widespread application has long been constrained by the limitations of traditional detection methods. Conventional hematoxylin-eosin (H&E) staining depends on the morphological interpretation of pathologists, which can be subjective. It often struggles to detect mucin “phenotypic drift,” especially in cases of incomplete intestinal metaplasia or early dysplasia, leading to variable diagnostic consistency. Immunohistochemistry provides molecular evidence and is considered a gold standard, but it has limitations, including complex workflow, high cost, long turnaround time, and increased use of precious tissue samples. These challenges are particularly relevant in large-scale screening and longitudinal monitoring of gastric precancerous lesions. Recent advances in artificial intelligence, especially deep learning combined with digital pathology, offer new approaches to address these limitations.

### 6.1. AI-Based Virtual Staining for Precise Identification of Mucin Phenotypes

**Figure 5:**
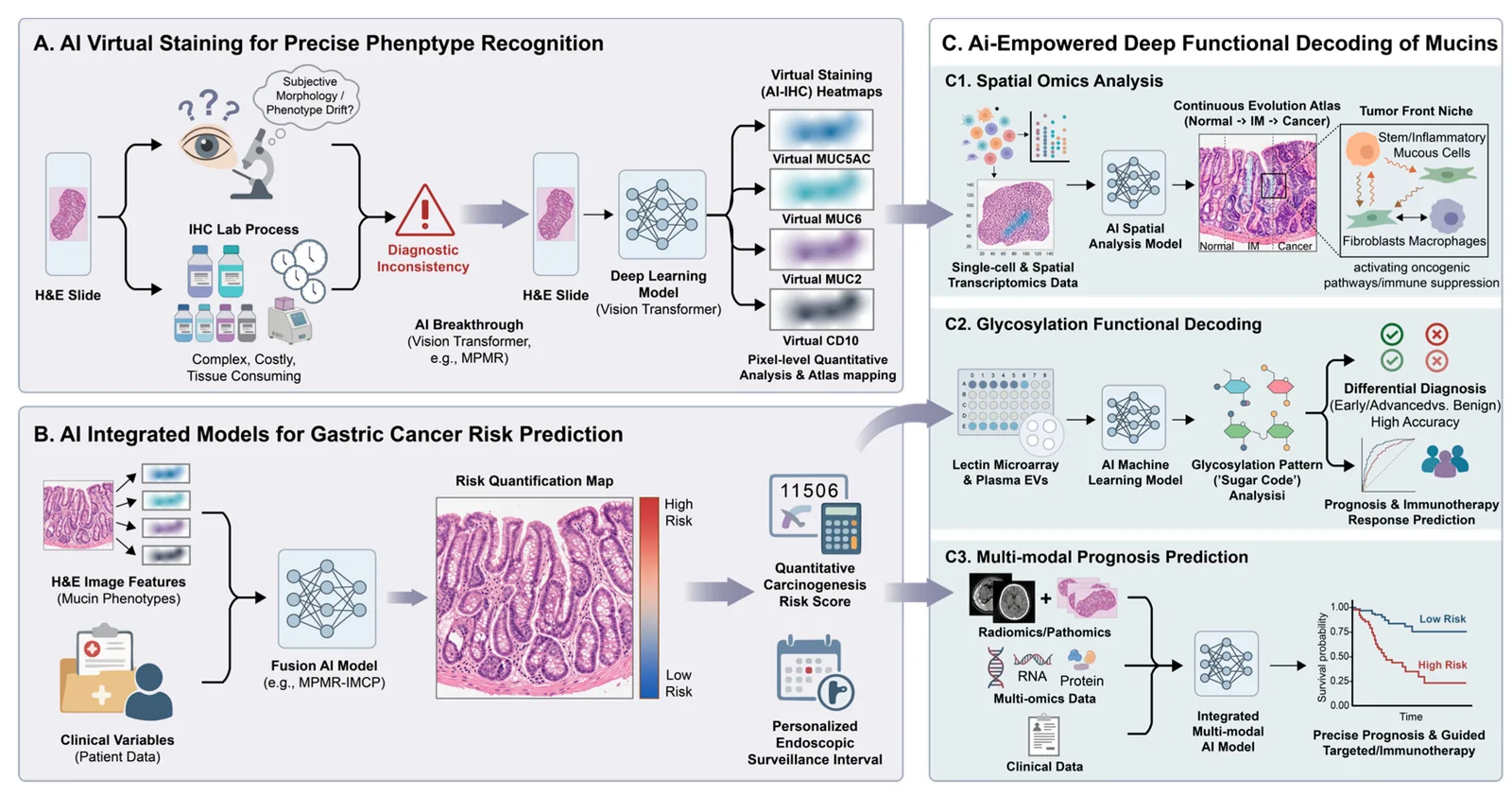
Precise identification of gastric cancer mucin phenotypes empowered by artificial intelligence. (**A**) AI-based virtual staining can predict MUC5AC, MUC6, MUC2, and CD10 expression from routine H&E images and generate simulated staining heatmaps. (**B**) AI-integrated risk models can combine mucin features with clinical variables to estimate malignant progression risk and support individualized endoscopic surveillance. (**C**) AI-enabled functional decoding can integrate spatial and single-cell omics, extracellular-vesicle glycan profiling, pathomics, and clinical data to investigate mucin-expressing cell states, glycosylation patterns, prognosis, and treatment response.

The core breakthrough of artificial intelligence in this field lies in its ability to mine “subvisual features” embedded in routine H&E slides that are beyond the resolution limit of the human eye, thereby enabling high-accuracy prediction of the expression of specific mucins or other molecular markers and generating visual heatmaps referred to as “virtual staining” or “AI-IHC.” The value of this technology lies not only in its ability to obtain molecular information in a “non-destructive” manner, but also in its capacity to perform pixel-level quantitative analysis and spatial mapping of expression patterns across whole tissue sections. A recent study reported that the MPMR model, built on a Vision Transformer architecture, was able to directly predict the expression of four key mucin markers, MUC5AC, MUC6, MUC2, and CD10, from H&E images. Its predictive performance achieved very high accuracy in independent validation, providing an efficient and cost-effective alternative for routine clinical mucin phenotype analysis [40] (Figure 5A).

### 6.2. AI-Integrated Models for Predicting Gastric Cancer Risk

Beyond virtual staining of mucin phenotypes, the deeper value of artificial intelligence lies in its ability to integrate multisource information and build integrated risk prediction models that go beyond single-marker detection, thereby transforming static molecular phenotypes into dynamic clinical decision-making tools. For example, the MPMR-IMCP risk model developed on the basis of the MPMR framework deeply integrates mucin phenotype imaging features extracted by deep learning with patient clinical variables, significantly improving the performance for predicting the risk of malignant transformation in intestinal metaplasia; its predictive ability was significantly superior to that of conventional models based only on clinical variables [40]. This model can quantitatively identify and precisely localize high-risk incomplete intestinal metaplasia regions—for example, by capturing the critical “mixed phenotype” feature of residual MUC5AC signals within MUC2-positive areas—and can then calculate a quantitative cancer risk score, thereby providing an objective and reproducible basis for determining individualized endoscopic surveillance intervals [40]. This technological breakthrough marks a shift in the management of gastric precancerous lesions from an experience-based model relying on rough morphological staging to a risk-quantified stratification model based on the deep integration of molecular phenotypes and artificial intelligence, achieving a transition from merely “seeing” molecular phenotypes to “understanding” their clinical significance (Figure 5B). Clinically, such models could support risk-adapted surveillance by integrating AI-derived mucin phenotypes with conventional risk factors. Patients with persistently higher predicted risk could be prioritized for high-quality endoscopy, targeted biopsies, and shorter surveillance intervals, whereas patients with consistently low predicted risk could be considered for less intensive follow-up, subject to prospective validation and guideline-defined thresholds. Serial reassessment could update risk estimates as mucin phenotypes evolve.

### 6.3. AI-Enabled Deep Decoding of Mucin Function

By enabling virtual staining of mucin phenotypes and supporting risk stratification, artificial intelligence extends its role from assessing static mucin expression to providing insights into their dynamic functions and clinical relevance. The biological functions of mucins depend not only on whether they are expressed, but are also finely regulated by their spatial distribution, cellular origin, and glycosylation status, and the powerful multimodal analytical capability of AI provides a breakthrough tool for precisely this purpose. At the level of spatial omics, recent studies have used AI-enabled single-cell transcriptomics and spatial transcriptomics to successfully delineate, within individual patients, a continuous evolutionary landscape from normal gastric mucosa through intestinal metaplasia to early gastric cancer, and have identified a critical transitional region located at the tumor front. This region is enriched with a subset of foveolar mucous cells with stem-like and inflammatory characteristics. Through specific signaling axes, these cells interact with fibroblasts and macrophages to jointly activate oncogenic pathways and promote the formation of an immunosuppressive microenvironment [82,83]. This finding not only reveals the central role of the heterogeneity of mucin-expressing cells in the origin of gastric cancer, but also elevates mucin research from the level of a single molecular marker to the spatiotemporal dynamic analysis of cellular interaction networks. At the level of glycosylation function, the pathological driving effect of mucins is highly dependent on their aberrant glycosylation, and AI is becoming a key engine for deciphering this “glycan code.” One study established a glycosylation pattern analysis platform for extracellular vesicles based on lectin microarrays, and used machine learning algorithms to model plasma samples from patients at different stages of gastric cancer, thereby achieving precise discrimination among early gastric cancer, advanced gastric cancer, and benign gastric diseases, with an accuracy of 92% [84,85]. Another study further developed a prognostic prediction model based on glycosylation patterns, with areas under the curve of 0.793, 0.914, and 0.988 for predicting survival at 200, 300, and 500 days, respectively, and showed excellent performance of 0.866–1.000 in predicting immunotherapy response [81]. These findings indicate that AI can convert microscopic changes in mucin glycosylation into quantifiable clinical indicators, which may facilitate liquid biopsy and longitudinal monitoring. For prognostic prediction, AI can integrate multimodal data to enhance the utility of mucin phenotypes. Models that combine multi-omics data have shown good performance in predicting responses to targeted therapies and combination immunotherapy. They can also stratify patients into different risk groups, supporting more individualized treatment decisions [86,87]. These studies demonstrate that AI can analyze mucin phenotypes in multiple dimensions. It can identify specific features and contextualize them within the tumor microenvironment, revealing associations with immune cells, stromal components, and therapeutic responses (Figure 5C). These findings suggest that AI-assisted mucin research is progressing from simple phenotype identification to interpreting functional roles, offering insights from the molecular to the systems level that may support precision diagnosis and treatment of gastric cancer. At the cancer stage, AI may also serve as a prescreening tool for biomarker-directed therapy. H&E-based models could prioritize cases for confirmatory testing when they predict tumor-associated MUC1 glycoforms or CLDN18.2 and could identify representative regions for pathological assessment. Recent multicohort evidence supports the feasibility of predicting CLDN18.2 expression from routine H&E slides [88]. However, treatment eligibility should not be determined by AI alone: CLDN18.2-targeted therapy requires confirmation with a validated immunohistochemical companion diagnostic, whereas MUC1-directed strategies remain investigational and require epitope- or glycoform-specific validation.

## 7. Conclusions and Future Perspectives

Mucin reprogramming occurs throughout the progression of gastric cancer. In normal gastric mucosa, MUC5AC and MUC6 establish regional barrier systems. During the precancerous stage, ectopic activation of transcription factors such as CDX2 drives lineage reprogramming, leading to the acquisition of MUC2 and loss of gastric-type mucins. In invasive gastric cancer, aberrant glycosylation contributes to functional remodeling and immune evasion. These changes link environmental exposures, including Helicobacter pylori infection, with genetic background and the malignant phenotype. Mucin expression profiles provide molecular criteria for distinguishing subtypes of intestinal metaplasia, classifying gastric cancer molecular subtypes, and determining tumor origin. This approach extends pathological evaluation from morphological observation to functional molecular pathology, facilitating early identification of high-risk lesions and individualized prognostic assessment. Understanding the mechanisms of mucin reprogramming has created opportunities for precision-targeted therapy. Examples include inhibitors targeting the intracellular domain of MUC1, antibody–drug conjugates directed against tumor-specific glycosylated epitopes, and interventions aimed at reversing abnormal epigenetic programming. These strategies support a shift from broad-spectrum chemotherapy to therapies targeting specific molecular vulnerabilities. Artificial intelligence has further enhanced diagnostic precision. By enabling automated, quantitative analysis of mucin phenotypes and glycosylation patterns, AI reduces interobserver variability and supports risk stratification and treatment decision-making. Overall, research on mucin reprogramming integrates basic biological insights with clinical practice, providing tools and frameworks for early diagnosis, targeted therapy, prognostic evaluation, and AI-assisted precision diagnostics.

Future research on mucin reprogramming is expected to integrate multiple dimensions along three main axes: mechanistic dissection, precise identification, and precision intervention. First, studies will increasingly employ multidimensional spatiotemporal analysis. Combining mucinomics with technologies such as spatial transcriptomics, single-cell sequencing, and metagenomics, researchers aim to construct dynamic maps of gastric carcinogenesis at subcellular resolution. This approach may clarify how different cell clones with distinct mucin phenotypes interact with stromal cells, immune cells, and microorganisms to shape tumor microenvironments. For example, it may help explain why gastric-type signet ring cell carcinoma with retained MUC5AC tends to disseminate to the peritoneum, whereas intestinal-type tumors expressing MUC2 more frequently metastasize to the liver, suggesting potential targets for interventions that block specific metastatic routes. Second, advances in glycomics and artificial intelligence are likely to drive clinical translation. Glycosylation determines the functional heterogeneity of mucins, so future studies will focus on decoding the “glycan code,” including truncated glycans such as Tn and STn, and specific patterns of sialylation and fucosylation. This knowledge may support the development of liquid biopsy biomarkers and guide targeted therapies, such as antibody–drug conjugates and therapeutic vaccines against glycosylated epitopes. AI, integrated with digital pathology, will support automated, quantitative analysis of mucin phenotypes and glycosylation patterns, reduce interobserver variability, and assist in risk stratification and treatment decisions. Key priorities include improving model interpretability; prospectively validating calibrated decision thresholds across centers; and establishing standardized human–AI workflows and reporting formats in which AI supports, rather than replaces, endoscopists, pathologists, and oncologists. Finally, systematic interventions targeting the underlying mechanisms of mucin reprogramming represent the next stage of precision therapy. The interacting mechanisms (microbiome dysbiosis, epigenetic dysregulation, and aberrant glycosylation) form the driving network of reprogramming. Therapeutic strategies are likely to evolve from single-target approaches toward coordinated multi-axis interventions. Vertical combinations may include sequential or simultaneous treatments targeting different axes, such as microbiome modulators, epigenetic regulators, and immune checkpoint inhibitors, to reverse reprogramming and improve the immune microenvironment. Horizontal selection involves choosing the dominant intervention based on the patient’s mucin-microbiome-epigenetic subtype. For instance, a highly glycosylated MUC1-type tumor may be treated with glycosylation inhibitors plus anti-MUC1 ADCs, whereas a dysbiosis-associated, epigenetically silenced tumor may benefit from microbiome modulation combined with low-dose epigenetic therapy. Challenges remain, including the development of selective and low-toxicity drugs, precise patient stratification using multi-omics data, and designing clinical trials to validate these complex combination strategies.

In summary, by integrating deep insights into molecular mechanisms, the empowerment of intelligent diagnostic tools, and the synergy of multiaxial targeted therapies, we may ultimately build a precision prevention and control system for gastric cancer based on mucin reprogramming and covering the entire disease course.

## Figures and Tables

**Table 1:** Classification, Chromosomal Localization, and Major Organ Distribution of Human Mucin Family Members.

Mucin	Type/Status	Chromosomal Location	Major Organs/Tissues of Distribution	Overview of Core Physiological Functions and Special Notes
MUC1	Membrane-bound	1q21	Glandular epithelia throughout the body (stomach, breast, lung, kidney)	An apical membrane signaling sensor involved in immune regulation and cell adhesion; frequently aberrantly expressed in tumors (e.g., CA15-3) [10,13].
MUC2	Secreted (gel-forming)	11p15.5	Small intestine, colon, trachea	Forms the structural framework of intestinal mucus, establishes the inner and outer mucus layers, and maintains symbiosis with the microbiota [18].
MUC3A/B	Membrane-bound	7q22	Small intestine, colon, gallbladder	Involved in intestinal epithelial repair and cell migration [19].
MUC4	Membrane-bound	3q29	Trachea, colon, cervix	A ligand for ErbB2 (HER2), regulating cell growth and differentiation [20].
MUC5AC	Secreted (gel-forming)	11p15.5	Stomach (foveolar epithelium), respiratory tract	A core component of the gastric surface anti-acid barrier and a major component of airway mucus [21].
MUC5B	Secreted (gel-forming)	11p15.5	Salivary glands, respiratory tract, cervix	Maintains the rheological properties of mucus and participates in airway clearance [22].
MUC6	Secreted (gel-forming)	11p15.5	Stomach (deep glands), duodenum	Protects fundic gland stem cells and exhibits anti-*H. pylori* activity [23].
MUC7	Secreted (soluble)	4q13.3	Salivary glands, respiratory tract	Does not form gels; has antifungal and antibacterial activity (e.g., binding to streptococci) [24].
MUC8	Secreted	12q24	Respiratory tract, uterus	Upregulated under inflammatory conditions; its function has not yet been fully clarified [25].
MUC9	Special (renamed)	1p13.2	Fallopian tube	Its current official name is OVGP1 (oviductal glycoprotein 1). It contains a mucin-like domain and is involved in fertilization and early embryonic development.
MUC10	Special (non-human gene)	N/A	Mouse salivary gland	Not present in the human genome. It is a mouse-specific gene, and its human direct ortholog is PROL1.
MUC11	Special (obsolete)	7q22	N/A	This designation has been discontinued. It resulted from an early sequencing error and is now confirmed to be part of the MUC12 gene sequence.
MUC12	Membrane-bound	7q22	Colon	Involved in the maintenance of intestinal homeostasis and is frequently downregulated in colorectal cancer [26].
MUC13	Membrane-bound	3q21	Intestine, lymphatic system	Protects the intestinal mucosa; overexpression is associated with inflammation and tumors [27].
MUC14	Special (atypical)	4q24	Vascular endothelium	Its current official name is EMCN (endomucin). It mainly participates in leukocyte–endothelial cell adhesion.
MUC15	Membrane-bound	11p14	Placenta, colon	Regulates trophoblast invasion; its function is still under investigation [28].
MUC16	Membrane-bound	19p13.2	Ocular surface, respiratory tract, peritoneal mesothelium	The largest mucin by molecular weight; contributes to formation of the glycocalyx barrier and is also the tumor marker CA125 [10,29].
MUC17	Membrane-bound	7q22	Small intestine, colon (especially the duodenum)	Stabilizes the apical membrane structure of intestinal epithelial cells and maintains barrier integrity [30].
MUC18	Special (reclassified)	11q23.3	Vascular endothelium, melanoma	Its current official name is MCAM or CD146. It actually belongs to the immunoglobulin superfamily (IgSF).
MUC19	Secreted (gel-forming)	12q12	Salivary glands, middle ear	Participates in defense of the oral environment and is less commonly expressed in the deep digestive tract [31].
MUC20	Membrane-bound	3q29	Kidney, colon	Regulates the Met signaling pathway and is associated with IgA nephropathy [32].
MUC21	Membrane-bound	6p21	Esophagus, lung	Masks cell-surface antigens and participates in immune evasion [33].
MUC22	Membrane-bound	6p21	Lung, colon	Has functional features similar to those of MUC21 and is associated with susceptibility to pulmonary diseases.

**Table 2:** Classification of mucin phenotypes in intestinal metaplasia.

Classifications	Mucin Subtype	Mucin Expression			
		MUC5AC	MUC6	MUC2	CD10
Normal Gastric Mucosa	Type 0	+	+	−	−
Complete Intestinal Metaplasia	Type I	−	−	+	+
Incomplete Intestinal Metaplasia	Type II/III	+	+	+	−

## Data Availability

No new data were created or analyzed in this study. Data sharing is not applicable to this article.
